# From the Colon to the Liver: How Gut Microbiota May Influence Colorectal Cancer Metastatic Potential

**DOI:** 10.3390/jcm13020420

**Published:** 2024-01-12

**Authors:** Irene Mignini, Giulia Piccirilli, Linda Galasso, Fabrizio Termite, Giorgio Esposto, Maria Elena Ainora, Antonio Gasbarrini, Maria Assunta Zocco

**Affiliations:** CEMAD Digestive Diseases Center, Fondazione Policlinico Universitario “A. Gemelli” IRCCS, Università Cattolica del Sacro Cuore, Largo A. Gemelli 8, 00168 Rome, Italy; irene.mignini@guest.policlinicogemelli.it (I.M.); giulia.piccirilli01@icatt.it (G.P.); linda.galasso0817@gmail.com (L.G.); fabrizio.termite01@icatt.it (F.T.); giorgio.esposto2@gmail.com (G.E.); mariaelena.ainora@policlinicogemelli.it (M.E.A.); antonio.gasbarrini@unicatt.it (A.G.)

**Keywords:** gut microbiota, colorectal cancer, liver metastasis, gut–liver axis

## Abstract

The gut microbiota’s influence on human tumorigenesis is a burning topic in medical research. With the new ontological perspective, which considers the human body and its pathophysiological processes as the result of the interaction between its own eukaryotic cells and prokaryotic microorganisms living in different body niches, great interest has arisen in the role of the gut microbiota on carcinogenesis. Indeed, dysbiosis is currently recognized as a cancer-promoting condition, and multiple molecular mechanisms have been described by which the gut microbiota may drive tumor development, especially colorectal cancer (CRC). Metastatic power is undoubtedly one of the most fearsome features of neoplastic tissues. Therefore, understanding the underlying mechanisms is of utmost importance to improve patients’ prognosis. The liver is the most frequent target of CRC metastasis, and new evidence reveals that the gut microbiota may yield an effect on CRC diffusion to the liver, thus defining an intriguing new facet of the so-called “gut-liver axis”. In this review, we aim to summarize the most recent data about the microbiota’s role in promoting or preventing hepatic metastasis from CRC, highlighting some potential future therapeutic targets.

## 1. Introduction

According to the GLOBOCAN estimates from the International Agency for Research on Cancer, colorectal cancer (CRC) is the third most commonly diagnosed neoplasia worldwide and the second highest cause of cancer-related mortality in both men and women [1]. The number of new cases is predicted to rise from 1.93 million in 2020, representing 10% of the global cancer incidence, to 3.2 million in 2040 [2]. Indeed, although its mortality is decreasing annually among older people (>50 years) who benefit from the large diffusion of screening programs including fecal occult blood tests and colonoscopy with the removal of precursor lesions, CRC incidence and mortality are still increasing among young people, especially in developing countries [1]. Thus, to face the rising incidence of early-onset CRC, in 2018, the American Cancer Society recommended beginning screening programs at the age of 45 instead of 50 years [3].

CRC metastases are the main cause of death, and the liver is the most affected organ due to the direct vascular connection between gut and liver represented by the portal vein system [4,5]. In 14% to 18% of patients, liver metastases are already present when the primary tumor is diagnosed, while in 10% to 25% of cases, they are identified at the time of CRC resection [6]. The 5-year survival rates are generally poor, reaching 14% when CRC is diagnosed at a metastatic stage [7]. Therefore, a deeper understanding of the mechanisms underlying the metastatic spread of CRC is of outmost importance to improve patients’ prognosis.

In recent decades, with the growing interest towards microbiota, the perspective on human physiological and pathological processes has been revolutionized: if a healthy body is nowadays considered as having a balanced interaction between its own eukaryotic cells and prokaryotic microorganisms living in different body niches, diseases are conceived as a result of the disruption of such a complex equilibrium [8]. Consistently, the role of microbiota in tumorigenesis, and particularly the gut microbiota, has been largely investigated, and a consensus statement focusing on microbiota’s influence on human carcinogenesis was published in 2019 by the International Cancer Microbiome Consortium [9]. In this document, the authors define dysbiosis—the alteration of the structure and functions of a “healthy” microbiota—as a cancer-promoting condition and identify five main mechanisms through which dysbiosis may impact tumor development, including inflammation, interaction with intracellular pathways, integration with the human genome, influence on immune-mediated anticancer surveillance, and interaction with multiple metabolites. Hence, the term “oncomicrobiome” has been introduced to describe the characteristic microbiota profile shared by oncologic patients, which is different from healthy individuals and may become a non-invasive tool for early diagnosis [10].

As for CRC, the first hypothesis that the gut microbiota can contribute to its initiation and development was formulated in 1997 by Dove et al., who observed that gut bacteria were necessary for the growth of intestinal adenomas in mice, while germ-free mice developed 2-fold fewer adenomas in the small intestine than controls [11]. In the following years, the effect of the gut microbiota on colorectal tumorigenesis has been a major field of research, regarding not only sporadic CRC but also CRC occurring in high-risk conditions such as inflammatory bowel diseases, thus underlying microbiota’s role both in the adenoma–carcinoma sequence typical of sporadic tumors and in the inflammation–dysplasia–carcinoma process which is characteristic of inflammatory conditions [12]. Different studies focused their attention on specific bacteria which may contribute to CRC development, including colibactin-producing *Escherichia coli*, *Bacteroides fragilis*, *Fusobacterium nucleatum,* and *Providencia*, together with a significant decrease in butyrate-producing bacteria such as *Roseburia* and *Fecalibacterium* [13,14,15,16,17,18,19,20].

Recently, research has also shed light on the role of the intestinal microbiota in the occurrence of CRC-associated liver metastasis [21,22]. Therefore, in this review, we aim to define an intriguing new facet of the so-called “gut-liver axis”, providing an overview of current data on microbiota’s influence on promoting or preventing CRC-derived liver metastasis. We first describe the main molecular mechanisms involved in metastasis development, specifically analyzing evidence about the most relevant bacterial species and their interaction with pro- or anti-carcinogenic human metabolites. Afterwards, we summarize how the microbiota may have an impact on patients’ response or resistance to antitumor systemic treatments, notably chemotherapy and immunotherapy. In the last paragraphs, we present available data on microbiota modulation as a potential therapeutic strategy, thus paving the way for future research studies and clinical applications.

## 2. Gut Microbiota and CRC Metastasis Development

The availability of modern laboratory techniques, notably 16s-ribosomal ribonucleic acid (rRNA) sequencing, shotgun analysis, and metagenomics, has provided a new insight into microbiota structure and functions in healthy and pathological conditions. Indeed, analysis of fecal samples using metagenomic shotgun sequencing from healthy individuals and patients with advanced adenoma or carcinoma revealed an higher prevalence of several bacterial species, including various *Bacteroides*, *Parabacteroides*, *Alistipes putredinis*, *Bilophila wadsworthia*, *Lachnospiraceae bacterium*, and *Escherichia coli*, in both carcinoma and adenoma patients [23].

Despite the complexity of microbiota–host interactions and the lack of exhaustive data, some mechanisms have been hypothesized through which microbiota may promote liver metastasis. A potential way may be bacterial migration through the portal vein system, which is limited in healthy subjects thanks to efficient tight junctions and the gut–vascular barrier. In CRC patients, gut–vascular barrier impairment has been observed, allowing intestinal bacteria to translocate from the bowel lumen to the liver through the portal circulation, the same vascular route responsible for the hematogenous dissemination of neoplastic cells. Gut bacteria from primary CRC, reaching the liver, lead to the formation of “premetastatic niches” that favor metastasis development [24,25]. Therefore, dysbiosis seems to involve not only intestinal bacteria but also the liver microbiota. Bacterial translocation may drive the remodeling of the liver microenvironment, including liver immune cells, notably Kupffer cells, which play a protective role against metastasis. Thus, as Yuan et al. proved in their study, bacterial with a negative impact on Kupffer cells, promote liver metastasis [26].

Nonetheless, the exact role of intestinal bacteria has not been completely elucidated yet. Each of the next subsections specifically focuses on one of the main microbes that have been shown to play a role in the CRC metastatic process, providing details about their mechanisms of action in the CRC metastatic process.

### 2.1. Fusobacterium nucleatum

*Fusobacterium nucleatum*, a Gram-negative bacterium well known for its pro-carcinogenic traits [27,28,29], has gained attention as a potential biomarker of CRC [30]. Its pro-tumorigenic activities stem from the actions of FadA, an adhesin enabling invasion into host epithelial cells and subsequent activation of Annexin A1 [31]. Annexin A1, in turn, modulates the WNT/β-catenin pathway, which is indicative of a poorer prognosis [32]. Studies, notably by Chen et al. [33], have highlighted *F*. *nucleatum*’s adverse impact on patients’ prognosis. Their results revealed a correlation between the downregulation of the METTL3 gene and poorer overall and disease-free survival. Additionally, *F*. *nucleatum*’s pro-carcinogenic mechanisms involve the activation of inflammatory cytokines [34,35] and concurrently reducing anti-inflammatory and antitumor cytokines, such as T cells and natural killer (NK) cells [36].

Recent attention has been given to the potential role of *F. nucleatum* in the development of liver metastases from primary colorectal tumors. Indeed, as shown by Sakamoko et al., *F. nucleatum* is present in hepatic metastases from CRC and induces modifications of the liver immune microenvironment [37]. Specifically, it leads to an increase in proinflammatory cytokines such as Tumor Necrosis Factor (TNF)-α, interleukin (IL)-6, IL-17A, IL-12, interferon (IFN)-γ, monocyte chemoattractant protein (MCP)-1, Eotaxin, chemokine ligand (CXCL)-1, and IL-9. While MCP-1, Eotaxin, IL-12, and IL-6 are indicative of chronic inflammation, IFN-γ seems to have a dual role, being both antitumorigenic [38] and implicated in CRC metastasis formation [39]. The increase in IL-17A and TNF-α induced by *F. nucleatum* not only promotes CRC metastasis formation but also decreases the cytotoxic activity of CD8+ T cells, leading to reduced antitumor activity [37]. Moreover, *F. nucleatum* reduces the innate immune response [40] by decreasing NK cells [41], CD3+, CD4+, and CD8+ T cells, as well as activating regulatory T cells (Treg) [42], posing a greater risk of attenuated antitumor immunity within the liver [43,44].

### 2.2. Bacteroides fragilis

*Bacteroides fragilis*, a Gram-negative anaerobic bacterium, plays a pivotal role in maintaining intestinal health [28]. However, not all *B*. *fragilis* strains exert such a beneficial effect. Recent studies have indicated that certain strains produce Bacteroides fragilis toxin (BFT) or fragilysin, a 20 kDa zinc-dependent metalloprotease toxin that binds to colonic epithelial cells, potentially triggering inflammatory responses, particularly in intra-abdominal infections and abscesses. These strains also appear to modulate immune reactions and facilitate the growth of colon tumors [45,46].

The pathogenicity of Enterotoxigenic Bacteroides fragilis (ETBF) is primarily linked to the secretion of BFT. This toxin cleaves E-cadherin, a tumor suppressor protein, enhancing intestinal barrier permeability and activating the WNT/β-catenin pathway, which is frequently activated in CRC [47]. Recent insights from a limited-scale study from Turkey further support the association between CRC ad ETBF, showing a higher prevalence among CRC patients compared to controls [48]. These findings underline the potential proinflammatory and oncogenic traits of these bacteria in the colon. *Bacteroides fragilis* colonization enhances an intense immune response of the colonic epithelium, particularly involving IL-17-producing CD4+ T cells (Th17). This results in the activation of the STAT-3 pathway, which is crucial for Th17 cell differentiation. Also, studies using antibodies against IL-17 and the IL-23 receptors have indicated a decrease in colon tumors in ETBF-colonized mice, confirming the involvement of IL-17 in ETBF-induced tumorigenesis [49].

Interestingly, Parida et al. utilized a syngeneic mammary intraductal model colonized by gut-derived ETBF to explore distant metastasis and immune modifications. ETBF prompted widespread inflammation, fostering the development of premetastatic sites in crucial organs and creating an environment of immune suppression. Such alterations increased cytokine levels, the infiltration of supportive tissue, and enhanced blood vessel formation, accelerating the early spread of metastasis to the liver and lungs [50]. These results are consistent with the “seed and soil” theory, proposing ETBF-induced models of metastasis that operate regardless of the primary tumor size or presence.

### 2.3. Streptococcus gallolyticus

*Streptococcus gallolyticus*, formerly called *Streptococcus bovis*, is a Gram-positive bacterium linked to various health conditions, including human infections such as endocarditis and bacteremia [51,52], as well as CRC [53]. A potential association between this bacterium and an increased susceptibility to CRC development has been hypothesized. *S. gallolyticus* may expedite the progression of CRC by activating the WNT/β-catenin pathway, boosting c-Myc and Cyclin D1 expression, and fostering cell proliferation [54]. Additionally, it incites the release of inflammatory cytokines (TNF-α, IL-6, IL-1β, IL-8) in CRC cell lines, establishing a proinflammatory microenvironment and leading to tumor advancement [55,56,57]. While the involvement of *S. gallolyticus* in the genesis of metastasis from CRC remains under investigation, there are still many aspects to explore. A subtle indication of its potential role in distant metastasis can be gleaned from the research conducted by Boleij et al. [58], where mildly increased levels of *S. gallolyticus* antigen RpL7/L12 antibodies were identified in advanced CRC patients displaying lymph node or distant metastasis.

## 3. Metabolites from the Gut Microbiota and CRC Metastatic Potential

The gut microbiota exerts its effects by not only directly acting on pro- or anti-carcinogenic molecules but also through multiple products of bacterial metabolism. Indeed, it plays a pivotal role in metabolizing dietary components and generating a spectrum of metabolites that profoundly influence the host’s physiological processes. Among these metabolites are short-chain fatty acids (SCFAs), bile acids, and secondary metabolites, which have drawn significant attention for their potential involvement in cancer progression, particularly in metastasis development.

Notably, SCFAs such as acetate, propionate, and butyrate exhibit diverse effects on host cells, with evidence suggesting their protective role against CRC and its metastatic spread [59,60]. In a recent study conducted by Li et al. on rats, it was demonstrated that butyrate treatment induces differentiation in CRC cells while simultaneously reducing their resilience to oxidative stress. Consequently, this drives a notable decline in the metastatic potential of rat CRC cells. Also, the authors highlighted the impact of butyrate treatment on downregulating integrins and initiating apoptotic cell death through the activation of Nuclear factor kappa-light-chain-enhancer of activated B cells (NFkB), influencing the compromised β1-integrin/focal adhesion kinase/PI 3-kinase pathway [61]. Furthermore, Gomes et al. have suggested that SCFAs might have an indirect impact on the liver through the modulation of immune responses, inflammation, and the behavior of cancer cells, thereby influencing the potential for metastasis. Their research demonstrated that interferences in propionate metabolism create a pro-aggressive pattern in breast and lung cancer cells, heightening their propensity for metastasis [62].

On the contrary, intestinal microbiota converts primary bile acids originating from the liver into secondary bile acids, and alterations in bile acid metabolism and in their composition have been associated with CRC. Certain bile acids, namely deoxycholic acid (DCA) and lithocholic acid [63], have been linked to the promotion of inflammation and oxidative stress, potentially contributing to cancer progression and metastatic spread. Interestingly, Pai et al. demonstrated that, even at low concentrations (5 and 50 microM), DCA significantly increased the tyrosine phosphorylation of β-catenin, prompting the expression of urokinase-type plasminogen activator and cyclin D1. This resulted in the increased proliferation and invasiveness of CRC cells, together with a notable decrease in the binding of E-cadherin to β-catenin [64].

**Figure 1:**
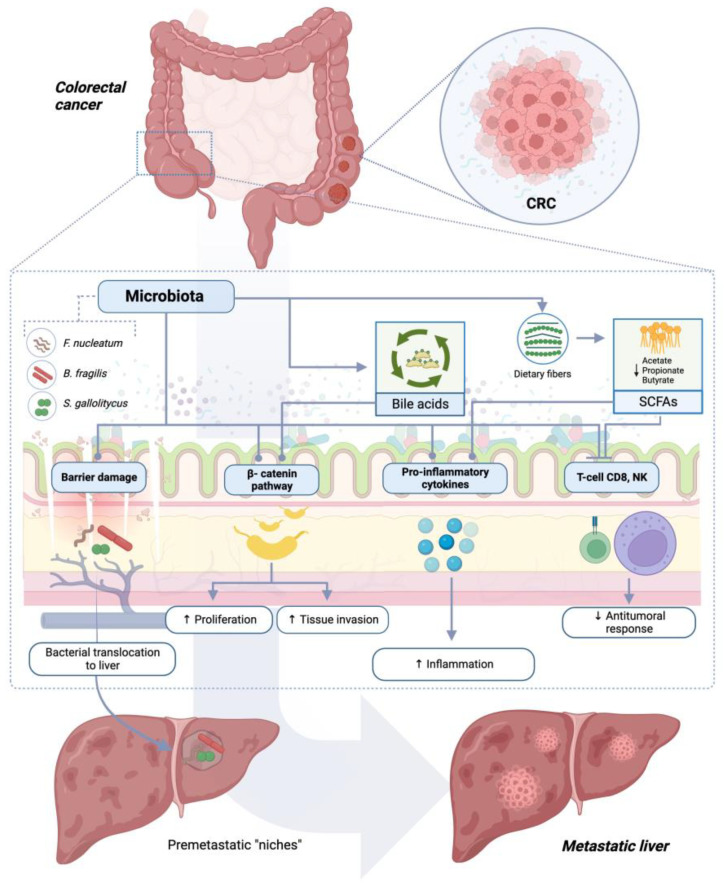
Gut microbiota impact on CRC liver metastasis. Intestinal bacteria may have an impact on CRC metastatic spread to liver either directly or through its metabolites, notably bile acids and short-chain fatty acids (SCFAs). Different molecular mechanisms are involved, encompassing the inhibition of anticancer immunosurveillance, upregulation of proinflammatory cytokines, and promotion of neoplastic tissue proliferation and invasiveness. Finally, gut barrier impairment associated with dysbiosis induces bacteria translocation to liver through portal circulation. Globally, all the aforementioned mechanisms increase CRC metastatic potential.

Figure 1 represents the main mechanisms through which the gut microbiota influences CRC liver metastasis development.

## 4. The Gut Microbiota and Response to Systemic Therapies

The treatment of metastatic advanced CRC is based on a combination of different systemic treatments, encompassing chemotherapy, targeted biologic agents, and novel immunotherapies [65]. The choice is complex and based on multiple factors, including the patient’s conditions and comorbidities, histology and molecular biology of the tumor, cancer location, and number of previous therapies [65]. However, the response rates to systemic therapies are heterogeneous among different patients due to the development of therapy resistance, which represents a major issue limiting clinical efficacy and patients’ survival.

The molecular bases underlying therapy resistance are still largely unknown, and the potential role of the gut microbiota is under investigation. Indeed, the gut microbiota, through its ability to metabolize drugs, may increase or decrease the toxicity of chemotherapeutics using enzymes and cellular mechanisms that influence how medications are activated or broken down. Microbial imbalance due to antibiotics severely affects the efficacy of chemotherapies involved in cancer treatment. In neoplastic conditions, especially during systemic treatment, the microbiota often loses its typical abilities of functional adaptation and plasticity: this may represent the missing link that explains, at least partially, why we observe such a wide range of patients’ outcomes in cancer treatment [66]. Recent studies demonstrated that the gut microbiota could interact and modulate the host response to chemotherapeutic drugs through three main mechanisms: the facilitation of drug efficacy, abrogation and compromization of anticancer effects, and mediation of toxicity. Consequently, the gut microbiota has to be considered in terms of customized cancer treatment strategies, based on the evidence from human, animal, and in vitro studies showing that gut bacteria are closely linked to the pharmacological effects of chemotherapies and novel targeted immunotherapies [67].

### 4.1. The Gut Microbiota and Chemotherapy

In CRC patients, chemotherapy is used as an adjuvant treatment after surgery or in advanced cases. A mutual relationship exists between chemotherapy and microbiota: while cytotoxic agents change bacterial composition promoting dysbiosis, the efficacy of the chemotherapeutics may be affected by the gut microbiota. For example, Viaud et al. investigated the role of intestinal commensal bacteria in mouse models treated with cyclophosphamide, an alkylating cytotoxic agent, and observed that this drug can disrupt the intestinal barrier with the subsequent translocation of commensal bacteria. Conversely, long-term treatment with antibiotics reduced the anticancer effect of cyclophosphamide, and mice receiving vancomycin and colistin became resistant to treatment, thus suggesting a role of microbiota in promoting the antitumor effect [68,69].

The main cytotoxic agents approved for CRC are platinum-derivatives, 5-fluorouracil (5-Fu), capecitabine, and irinotecan, combined with targeted therapies against epidermal growth factor receptor (anti-EGFR) or vascular endothelial growth factor (anti-VEGF) [65]. The influence of commensal bacteria on the response to platinum therapy has already been described by Iida et al., who noticed that the disruption of microbiota impairs the response to platinum in mouse models which received a subcutaneous injection of neoplastic cells. In order to develop an adequate response to cancer therapy, instead, an intact gut microbiota was necessary to modulate the tumor microenvironment and immune cell functions [70].

In contrast to the beneficial role of the gut microbiota in promoting chemotherapy’s effects in animal models, the idea that microbiota could inhibit the efficacy of such therapies, leading to antitumor treatment resistance, was unexpected. Recent studies have concentrated particularly on *F. nucleatum*, a well-known cancer-promoting microbe, whose abundance gradually increases from normal tissues to adenoma and adenocarcinoma tissues [26,27,67]. *F. nucleatum* is also abundant in patients with CRC recurrence post-chemotherapy. Yu et al. found that *F. nucleatum* promoted CRC resistance to chemotherapeutics such as oxaliplatin and 5-Fu, targeting Toll-like receptor 4 (TLR-4) and MyD88 innate immune signaling and specific microRNAs (miRNA-18a* and miRNA-4802) to activate the autophagy pathway and alter CRC’s chemotherapeutic response. Overall, the results of this study support a model in which *F. nucleatum* rearranges cancer cells to survive chemotherapy [43]. Another contribution to the potential role of *F. nucleatum* as an inducer of 5-Fu chemoresistance of CRC cells comes from Zhang et al., who demonstrated that the abundance of *F. nucleatum* correlates with chemoresistance in advanced CRC patients who received standard 5-Fu-based adjuvant chemotherapy after radical surgery. They found that *F. nucleatum* is implicated both in vitro and in vivo in the upregulation of BIRC3, a member of the inhibitor of apoptosis proteins (IAPs) responsible for the reduced chemosensitivity of CRC cells to 5-Fu [71]. The IAP family includes several important molecules involved in apoptosis, characterized by the presence of a ~70 amino acid called the Baculovirus IAP Repeat (BIR) domain, which is important for the binding and inhibition of caspases [72,73]. BIR mediates protein recognition and protein–protein interactions [74]; BIRC3 displays anti-apoptosis properties by directly inhibiting the caspase cascade, contributing to chemoresistance in malignancies, including CRC [75]. Zhang et al. pointed out that BIRC3 was the most upregulated gene induced by *F. nucleatum* in infected CRC cell lines, making *F. nucleatum* and this gene promising therapeutic targets for reducing chemoresistance to 5-Fu treatment in advanced CRC [71].

Other intestinal bacteria, such as *Streptococcus bovis*, *Enterotoxigenic Bacteroides fragilis,* and *Enterococcus faecalis,* have been reported as capable of promoting the occurrence, development, and chemoresistance of CRC through inflammatory reaction, genotoxins, oxidative stress, metabolites, and biofilms [76]. *Peptostreptococcus anaerobius*, an anaerobic Gram-positive bacterium that can be normally found in human oral cavities and intestinal tracts, has been found in high abundance in the intestinal flora of chemoresistant CRC patients [77,78]. Gu and colleagues observed that *P. anaerobius* accumulation in tumor lesions could mediate the recruitment of myeloid-derived suppressor cells (MDSCs) into the CRC microenvironment, promoting IL-23 secretion and finally leading to epithelial–mesenchymal transition (EMT) and the chemoresistance of CRC cells to oxaliplatin. These results were achieved both in vitro and in vivo. In CRC mouse models treated with *P. anaerobius*, the bacterium activated tumor-associated myeloid cells (TMCs), enhancing them to produce higher levels of cytokine IL-23 than controls [79]. MDSCs are directly recruited from immature myeloid cells by tumor-derived growth factors and inflammatory factors to modulate the immune response and promote CRC progression [80,81], and high levels of MDSCs have been associated with worse patient outcomes [82]. To summarize, the colonization of *P. anaerobius* in CRC lesions mediates the recruitment of MDSCs into the tumor microenvironment; there, MDSCs secrete tumor-promoting cytokines, especially IL-23. The subsequent crosstalk with the surrounding cells promotes chemoresistance.

### 4.2. Gut Microbiota and Immunotherapy

Since cancer progression is associated with immunosurveillance failure, therapies tend to stimulate the immune system to eliminate malignant cells [83]. Consequently, long-term responses to immunochemotherapies generally rely on the activation or reactivation of anticancer immune responses [84,85,86]. A therapeutic revolution has come with the development and regulatory approval of immunotherapies, especially immune checkpoint inhibitors (ICIs). It has been observed that immunotherapy is capable of eliciting a durable clinical response and also long-term remission [87]. Among ICIs, the first immuno-oncologic target to be approved was the cytotoxic T lymphocyte protein 4 (CTLA-4) [88]. Since then, several different antibodies have been approved such as anti-programmed cell death protein 1 (anti-PD-1) or anti-programmed cell death 1 ligand 1 (anti-PD-L1). These therapies act using antibodies to prevent the interaction of certain proteins (for example CTLA-4) with their ligands, triggering an otherwise ineffective immune response against tumors [89]. Immunotherapies have been demonstrated to be effective both alone or combined with other antitumor therapies [90], and their application has been extended on a wide range of cancers [56,57,58].

It has been observed that in patients affected by different tumors, such as melanoma, non-small-cell lung cancer, and urothelial and renal cell carcinoma, liver metastases reduce systemic antitumor immunity and significantly diminish clinical benefits from immunotherapy, regardless of disease histology. To explore how liver metastases affect the response to immunotherapy, Yu et al. established a preclinical model, demonstrating that liver metastases regulate CD8+ T cell activity by recruiting immunosuppressive macrophages that promote antigen-specific T cell apoptosis within the liver. This results in the systemic loss of antigen-specific T cells. This work pointed out that the presence of liver metastases could represent a potential negative baseline determinant of immunotherapy response [91]. Other studies also showed the role of radiotherapy to be to enhance immunotherapy efficacy both in patients [92,93,94] and preclinical models [95,96], demonstrating that liver-directed radiotherapy reshapes the liver immune microenvironment in order to prevent antigen-specific T cells loss and restores immunotherapy efficacy in models of liver metastases.

Recent findings propose that liver involvement also confers resistance to ICI treatment in advanced CRC. For example, Cohen et al. reported that liver involvement is a poor prognostic factor [97]. Fakih et al. showed that the median overall survival, progression-free survival, and overall response rate in patients with liver metastasis were lower compared with non-metastatic CRC treated with nivolumab and regorafenib [98]. Chen et al. conducted a randomized controlled trial to investigate whether the presence of liver metastasis is an indicator of treatment resistance to ICIs in advanced CRC. Their findings were consistent with previous results; patients without metastases had improved outcomes, regardless of treatment [99].

In CRC, testing for cancer mutations is crucial to choose the most appropriate systemic therapy. Some forms of CRC show high microsatellite instability (MSI-H), which leads to an accumulation of genetic errors such as deletion and insertion mutations at simple repeated sequences. This occurs during DNA replication as a consequence of defects in the mismatch repair system [100]. Subsequently, genomes of cancers deficient in mismatch repair (dMMR) contain exceptionally high numbers of mutation-associated neoantigens that might be recognized by the immune system [101]. Current guidelines recommend monoclonal antibodies against PD-1/PDL1 (nivolumab or pembrolizumab) as a first-line therapy in metastatic CRC with dMMR/MSI-H status [65]. However, patients with dMMR/MSI-H CRC account for 5% of all metastatic CRC, while the large majority of patients have a microsatellite-stable (MSS) disease that does not benefit from anti-PD-1/PD-L1 therapy. Researchers are trying to understand the etiopathogenesis of immunotherapy resistance and the gut microbiota has been proposed as a potential link, although the exact molecular mechanisms are still unknown.

Jiang et al. found that metastatic CRC patients who do not respond to anti-PD1 had a greater abundance of *F. nucleatum* and increased succinic acid. They ascribed the tumor-suppressive effect to the capacity of *F. nucleatum*-derived succinic acid to interfere with the cGAS-IFN-β pathway, which is crucial in CD8+ T cell trafficking to the tumor microenvironment, consequently causing a reduction in the antitumor response. In a mouse model, they used fecal microbiota transplantation (FMT) from responders with low *F. nucleatum* to confer sensitivity to anti-PD1 mice. Furthermore, antibiotic treatment with metronidazole reduced the intestinal abundance of *F. nucleatum*, with a consequent reduction in serum succinic acid levels so that the tumor could be sensitized again to immunotherapy in vivo. These findings indicate that tumor resistance to immunotherapy can be induced by the gut microbiota, which creates an intense crosstalk in the tumor microenvironment in CRC [102]. Song et al. investigated the impact of lipopolysaccharide (LPS) on CRC immunotherapy, finding that LPS is abundant in CRC cells and is associated with low responses to anti-PD-L1 therapy. They also noticed that, after administering bactericidal treatment to clear Gram-negative bacteria from the gut, LPS levels were reduced and T cell infiltration into cancer tissue increased. To further support their findings, they built-up an engineered molecule, more specifically a fusion protein targeting LPS, to block LPS inside the tumor. This LPS trap system significantly boosted the effect of anti-PD-L1 therapy and demonstrated an ability to attenuate CRC liver metastasis, supporting the importance of blocking LPS in the gut–liver axis [103].

However, microbiota do not always promote pharmacological resistance. Conversely, some bacteria, such as *Bacteroidales* [104], *Bifidobacterium* [105,106,107], or *Akkermansia muciniphila* [108,109], have proved able to stimulate the anticancer effect of therapies in different types of neoplasias.

Mager et al. explored the efficacy of ICIs in mice with azoxymethane/dextran sulfate sodium-induced colitis-associated cancer and observed that some bacteria were able to significantly enhance the efficacy of anti-PD-L1 and anti-CTLA-4. In this study, they isolated three bacterial species, which have been found in abundance in previously ICI-treated-tumor mice that responded to therapy: *Bifidobacterium pseudolongum*, *Lactobacillus johnsonii*, and *Olsenella* species. The isolation and identification of distinct bacterial species associated with ICI responsiveness provided the opportunity to identify the molecular mechanism involved in bacterial capacity to promote ICI efficacy. More specifically, they demonstrated that *B. pseudolongum* produces inosine, which is able to modulate the immunotherapy response. In contrast, although *L. johnsonii* did not produce inosine, it has been found to produce a large amount of hypoxanthine, which is a potent ligand of the same receptor as inosine. Finally, they conclude that the ICI-promoting effect of *B. pseudolongum* and *L. johnsonii* was mediated by inosine and hypoxanthine and collectively was dependent on T cell expression of the adenosine A2A receptor [110]. The discovery of a previously unknown microbial metabolite that can enhance immunotherapy may be utilized to develop microbial-based adjuvant therapies. Interestingly, also *F. nucleatum* should be included among the beneficial bacteria that promote immunotherapy effects. Indeed, Gao et al. revealed an alternative role for *F. nucleatum* in improving the therapeutic outcome in CRC. They demonstrated that the presence of this bacterium enhances the antitumor response to anti-PD-1/PD-L1 by inducing PD-L1 expression [111]. Such a surprising finding may be explained considering the hypothesis suggested by Hamada et al., who proposed that *F. nucleatum* action on immune response may differ basing on MSI status. According to their results, *F. nucleatum* was positively associated with tumor-infiltrating lymphocytes in MSI-high cancers, while there was a negative association in MSI-low cancers [112]. *Lactobacillus gallinarum*¸ instead, seems to improve anti-PD-1 efficacy in both MSI-high and MSI-low CRC tumors by suppressing the intratumoral infiltration of Treg and enhancing effector function of CD8+ T cells, as described by Fong et al. *L. gallinarum*-derived indole-3-carboxylic acid was identified as the functional metabolite capable of modulating antitumor immunity by antagonizing the activation of a pathway that leads to Treg differentiation [113].

In conclusion, these achievements indicate that the gut microbiota could be a new therapeutic target to improve CRC immunotherapy response.

Studies investigating the gut microbiota influence on systemic treatment response in metastatic CRC are summarized in Table 1.

## 5. Microbiota-Based Therapies: Gut Microbiota Modulation

Considering the growing role being recognized for the gut microbiota in liver metastasis development from CRC, a new challenge to be pursued is represented by targeting intestinal dysbiosis in order to select the bacterial communities which may be protective against tumor spread or may better influence response to treatment. Microbiota modulation appears as a promising add-on strategy to reduce metastatic risk and boost anticancer drugs’ benefits.

### 5.1. Probiotics

Probiotics have been proposed as novel therapeutic agents, since different probiotics may potentially inhibit CRC by different mechanisms, although their efficacy on CRC is not yet completely defined. They can downregulate inflammation and reduce the accumulation of carcinogenic metabolites to prevent CRC, regulate the immune system, and inhibit the progression of CRC [114]. Moreover, probiotics can be engineered to shift from a proinflammatory profile to an anti-inflammatory one via genetic modification or protein deletion. For example, the deletion of the phosphoglycerol transferase gene in *Lactobacillus acidophilus* suppresses the expression of lipoteichoic acid, an immunostimulatory protein. The oral administration of deleted *L. acidophilus* causes the downregulation of proinflammatory mediators and reduces colonic inflammation and colonic polyp development [115,116]. Hence, probiotic engineering has been proposed as an alternative strategy to achieve the desired immunomodulatory effect, since modified probiotics are able to improve the intestinal barrier function via SCFAs, secrete antioxidant and anticancer compounds, reduce the accumulation of carcinogenic metabolites to prevent CRC, produce anti-inflammatory factors, and regulate the immune system in order to inhibit the progression of CRC [114,117].

Different probiotics have been tested in CRC. The exact mechanisms of action are unknown, but a few studies have suggested several possible pathways. Aindelis et al. demonstrated that oral administration of probiotic *Lactobacillus casei*/*Lacticaseibacillus casei* is responsible for increased immune response in CRC and enhanced tumor infiltration by CD8+ T cells, resulting in a reduction in tumor growth [118]. In a study conducted by Chang et al., oral probiotic *Lactobacillus casei*/*Lacticaseibacillus casei* variety *rhamnosus* was shown to prevent intestinal mucositis induced by the folinic acid–fluorouracil–oxaliplatin (FOLFOX) regimen. The mechanism of action might involve modulation of the gut microbiota and the suppression of proinflammatory responses with the suppression of intrinsic apoptosis in intestinal injury [119]. Chen et al. focused on the modulation of probiotic *Clostridium butyricum* on the Wnt/β-catenin signaling pathway, through which a reduction in CRC growth was achieved [120]. Also, in CRC mouse models, it has been demonstrated that treatment with *Clostridium butyricum* and *Bacillus subtilis* can decrease cancer incidence, due to a decrease in the number of Th2 and Th17 cells, thereby inhibiting CD4+ and CD8+ T lymphocytes, blocking the cell cycle, reducing the secretion of inflammatory factors such as NFκB and IL-22, and promoting tumor cell apoptosis [121]. Furthermore, Walia et al. showed that probiotics have the potential role of lowering the expression of cyclooxygenase-2, which promotes tumor angiogenesis, thus downregulating tumor incidence [122].

Current available evidence only derives from preclinical studies and mostly describes probiotics’ effect on reducing the risk of primitive CRC development or improving treatment benefits. Specific data about liver metastasis, as well as data from clinical trials, are still poor. However, an interesting contribution supporting the use of probiotics in CRC treatment has been recently provided by Jakubauskas et al., who used a rat model to test probiotics’ effect on CRC liver metastasis. They evaluated the role of a probiotic mixture (*Lactobacillus casei*/*Lacticaseibacillus casei* W56; *Lactobacillus acidophilus* W37; *Lactobacillus brevis*/*Levilactobacillus brevis* W63; *Lactococcus lactis* W58; *Bifidobacterium lactis* W52; *Lactococcus lactis* W19; *Lactobacillus salivarius* W24; and *Bifidobacterium bifidum* W23), achieving pioneering results: such a probiotic mixture, administered daily, even without the addition of FOLFOX chemotherapy, induced a significantly reduction in angiogenesis and inhibition of CRC liver metastasis growth. The most important finding is that probiotic supplementation leads to tumor volume reduction by reducing tumor microvasculature [123].

The role of probiotics has also been investigated as add-on therapy with immunotherapy. Thus, Zhuo et al. evaluated the efficacy of *Lactobacillus acidophilus* lysates together with anti-CTLA4 and found that the combined therapy protected mice against CRC development and against dysbiosis, thus confirming the role of the gut microbiota in boosting immunotherapy, as discussed above [124].

Studies demonstrating the beneficial effects of probiotics on CRC are summarized in Table 2.

### 5.2. Fecal Microbiota Transplantation

FMT is a method consisting of the transplantation of fecal matter from healthy people into the intestinal tract of recipients to restore gut microbiota diversity. Currently, FMT has been approved for recurrent *Clostridium difficile* infections, while its application in other gastrointestinal and extraintestinal disorders is still a matter of study. Several clinical trials based on FMT are ongoing for a wide range of diseases, from inflammatory bowel diseases to cancers to psychiatric conditions [125]. In comparation with other microbiota-modulating strategies, FMT seems to confer multiple benefits: it has been utilized to improve bacterial, viral, fungal, or archaeal diversity into the recipient without disrupting the microbial gut ecology (as occurs during antibiotic treatments), and it can also be designed as a single-dose regimen; furthermore, FMT’s therapeutic benefits last longer than probiotics and prebiotics, whose colonization appears to be transient [126].

In the oncological field, FMT’s role has been especially investigated in combination with systemic therapies, and it seems to provide benefits both in enhancing treatment efficacy and in preventing or improving gastrointestinal side effects, notably colitis, which is frequently associated with chemotherapy or immunotherapy [127]. Chang et al. administered oral FMT in mice implanted with CRC cells and receiving FOLFOX chemotherapy, aiming to assess the effect on mucosal injury. They noticed that FMT reduced diarrhea and attenuated FOLFOX-induced inflammatory response. In particular, it decreased the number of apoptotic and NFkB-positive cells and the expression of TLR, MyD88, and IL-6 and restored gut microbiota composition [128].

Recently, some promising papers about FMT in oncologic patients have begun to appear in the literature, even if data on CRC are still anecdotal. At the moment, most data focus on the role of FMT as a potential strategy to overcome resistance to ICIs in patients with refractory melanoma. Routy et al. conducted a multicenter phase I trial combining FMT from healthy donors with nivolumab or pembrolizumab in a group of untreated patients with advanced melanoma, demonstrating that FMT is safe in a first-line setting. Furthermore, they confirmed the increasing anti-PD-1 efficacy obtained by microbiota modulation in a mice model [129]. Other clinical trials by Baruch et al. [130] and Davar et al. [131] observed good tolerance for the combination (FMT + ICI), clinical benefit in a subset of treated patients, and the induction of durable microbiota changes, with an increased abundance of taxa previously shown to be associated with response to anti-PD-1, increased CD8+ T cell activation, and decreased frequency of IL-8-expressing myeloid cells, which are involved in immunosuppression. Collectively, these studies provide evidence for the ability of FMT to affect immunotherapy response in patients affected by advanced melanoma.

Concerning CRC, Cheng et al. reported the case of a 57-year-old Chinese patient with dMMR CRC and liver metastasis, who received as third-line treatment a combination of bevacizumab, an anti-VEGF, plus tislelizumab, an investigative anti-PD1, plus oral gut microbiota capsules. The treatment consisted of eight cycles every 3 weeks, and FMT was administered at a dose of one capsule/day for the first four days. The patient achieved a partial response, allowing microwave ablation on liver lesions and hemicolectomy for the primary malignancy and, after surgery, a pathological complete response was reached [132]. Some clinical trials are also ongoing. Recently, data from a Chinese phase II trial have been published: RENMIN-2015. It is an open-label, single-arm study conducted on patients with MSS metastatic CRC, aiming to assess the efficacy and safety of a new drug combination as third-line or above therapy: FMT plus tislelizumab plus fruquitinib, a tyrosine kinase inhibitor of VEGF. FMT was administered as oral capsules at a dose of 30 capsules/day for 3 days in 3-week cycles. Of the 20 patients enrolled, 14 had liver metastasis, which represented a negative prognostic factor. The mean progression-free survival (PFS) was 9.6 months and, considering a PFS > 6 months as an index of treatment response, 12 patients were classified as responders, while 8 were non-responders. Side effects were common, but the global toxicity profile was manageable [133]. Another phase II clinical trial is currently ongoing (NCT04729322), aiming to assess the effect of FMT in combination with the re-introduction of anti-PD-1 therapy (Pembrolizumab or Nivolumab) in patients with metastatic dMMR CRC who had not previously responded to anti-PD-1 [134]. Similar studies are of primary importance to transfer in clinical practice knowledge acquired so far from preclinical research.

## 6. Conclusions

The pathogenetic mechanisms underlying liver metastasis growth from CRC are complex. The gut microbiota has proved to play a multifaceted role, although current evidence mainly derives from preclinical studies and results are at the first stage and suggestive. Further investigations are required to achieve a deeper understanding of such an intriguing aspect of the “gut-liver axis”. Intestinal bacteria seem to influence the gut barrier, inflammation processes, tissue proliferation, and anticancer immunosurveillance, as well as potentially interfering with systemic chemo-immunotherapies. Hence, developing efficient strategies to modulate the gut microbiota represents a research and clinical challenge to prevent CRC metastatic spread and improve patients’ outcomes.

## Figures and Tables

**Table 1:** Gut microbiota influence on systemic treatment response in metastatic CRC: involved bacteria and mechanisms of action. 5-Fu: 5-fluorouracil; PDL1: programmed cell death 1 ligand 1; PD1: programmed cell death protein 1; CTLA4: cytotoxic T lymphocyte protein 4; TLR: Toll-like receptor; EMT: epithelial–mesenchymal transition; MDSCs: myeloid-derived suppressor cells; LPS: lipopolysaccharide.

Reference	Drug	Effect on Treatment Response	Mechanism of Action	Involved Bacteria
Yu et al. (2017) [43]	Oxaliplatin 5-Fu	Resistance	Activation of the autophagy pathway targeting TLR4, MyD88, miR-18a, and miR-4802	*Fusobacterium nucleatum*
Zhang et al. (2019) [71]	5-Fu	Resistance	Inhibition of apoptosis through BIRC3 upregulation	*Fusobacterium nucleatum*
Gu et al. (2023) [79]	Oxaliplatin	Resistance	Promotion of EMT through recruitment of MDSCs	*Peptostreptococcus anaerobius*
Jiang et al. (2023) [102]	Anti-PD1	Resistance	Interaction with CD8 T cell trafficking to tumor microenvironment through succinic acid	*Fusobacterium nucleatum*
Song et al. (2018) [103]	Anti-PDL1	Resistance	LPS production	*Gram negative bacteria*
Mager et al. (2020) [110]	Anti-PDL1 Anti-CTLA4	Sensitivity	Activation of A2A inosine receptors through inosine and hypoxanthine production	*Bifidobacterium pseudolongum* *Lactobacillus johnsonii* *Olsenella*
Gao et al. (2021) [111]	Anti-PD1	Sensitivity	Induction of PDL-1 expression	*Fusobacterium nucleatum*
Fong et al. (2023) [113]	Anti-PD1	Sensitivity	Suppression of intratumoral infiltration of Treg and enhancement of CD8+ T cell functions	*Lactobacillus gallinarum*

**Table 2:** Probiotics’ beneficial role in colorectal cancer (CRC). FOLFOX: folinic acid–fluorouracil–oxaliplatin; CTLA4: cytotoxic T lymphocyte protein 4.

Reference	Model	Involved Bacteria	Administration Route	Effect
Aindelis et al. (2020) [118]	Preclinical (mice)	*Lactobacillus casei*/*Lacticaseibacillus casei*	Oral	Reduces tumor growth by increasing antitumor immune response
Chang et al. (2018) [119]	Preclinical (mice)	*Lactobacillus casei*/*Lacticaseibacillus casei* variety *rhamnosus*	Oral gavage	Prevents FOLFOX-induced mucositis
Chen D et al. (2020) [120]	Preclinical (CRC cells and mice)	*Clostridium butyricum*	Oral gavage	Inhibits tumor development
Chen ZF et al. (2015) [121]	Preclinical (CRC cells and mice)	*Clostridium butyricum, Bacillus subtilis*	Oral	Decreases tumor incidence
Walia et al. (2015) [122]	Preclinical (rats)	*Lactobacillus plantarum*/*Lactiplantibacillus plantarum* and *Lactobacillus rhamnosus*/*Lacticaseibacillus rhamnosus* GG	Oral gavage	Decreases angiogenesis and tumor incidence
Jakubauskas et al. (2022) [123]	Preclinical (rats)	Probiotic mixture (*Lactobacillus casei*/*Lacticaseibacillus casei* W56; *Lactobacillus acidophilus* W37; *Lactobacillus brevis*/*Levilactobacillus brevi* W63; *Lactococcus lactis* W58; *Bifidobacterium lactis* W52; *Lactococcus lactis* W19; *Lactobacillus salivarius* W24; and *Bifidobacterium bifidum* W23)	Oral gavage	Inhibits CRC liver metastasis growth
Zhuo et al. (2019) [124]	Preclinical (mice)	*Lactobacillus acidophilus* lysates	Intragastric	Enhances anti-CTLA4 activity

## Data Availability

No new data were created or analyzed in this study. Data sharing is not applicable to this article.
